# Are We Heading into a Dead End, or Are We Treading Water?

**DOI:** 10.3390/toxins18020088

**Published:** 2026-02-09

**Authors:** Wolfgang H. Jost

**Affiliations:** 1 Parkinson-Klinik Ortenau, 77709 Wolfach, Germany; w.jost@parkinson-klinik.de; Tel.: +49-7834-971-212; Fax: +49-07834-971-340; 2 Department of Neurology, University of Saarland, 66421 Homburg, Germany

The introduction of botulinum neurotoxin (BoNT) to our therapeutic strategies has been an incredible success story. This did not actually entail introducing a totally new drug, but was rather the result of interested physicians recognizing and using BoNT as a therapeutic option. Initially, this drug was used for hemifacial spasm, blepharospasm [1,2], and cervical dystonia [3]. Almost simultaneously, BoNT began to be used for spasticity [4] as well as for cosmetic purposes [5]. Later, many additional indications began to be treated with this drug, such as writer’s cramp, migraine, pain, and, most recently, sialorrhea. Of course, the available botulinum toxins were also improved upon and new preparations were introduced over time. Admittedly, in the early days, our current scientific standards were not yet taken in consideration, and trial-and-error played a substantial role then, but this approach also led to the development of ideas and approaches that might otherwise have been overseen. In this context, the empirical results of Justinius Kerner should also be considered, as he anticipated future therapeutic strategies; they can, in some respects, be regarded as a source of inspiration for us today [6]. Don’t get me wrong, I am of course not suggesting that unscientific methods are advantageous; I simply want to illustrate the development of BoNT therapy so that these developments can be understood from today’s perspective. It is possible that further lessons can be learned for the future.

I had my first experiences with botulinum toxin when I was searching for a substance for temporary muscle paralysis as part of my doctoral dissertation and found it in BoNT [7]. I began my neurology residency in 1989 and was able to participate in introducing BoNT at a university hospital, at a time when users were very innovative and sometimes even overly enthusiastic as well. Considering the time this all took place, the pressing question now is whether we have already discovered all the potential indications, or whether the hurdles for approval of new indications have become just too high. Should we, as in the 1990s, take the initiative ourselves again and not wait for approval studies from the industry? Interestingly, the last approval for a new indication was for sialorrhea [8]. All ensuing studies merely focused on improving the injection technique, classifying the disease symptoms, or expanding the approval of existing indications, e.g., spasticity in children and adults. But even with sialorrhea, it took 20 years from the first publication [8,9] until final approval was granted, because the indication was not economically viable enough for the manufacturers.

I am reaching the end of my professional career and I am somewhat afraid that if we are no longer courageous and innovative, we will lose our young talents. When we introduced ultrasound, we quickly saw a surge in the interest and motivation of our young colleagues. We urgently need that momentum again. This can be achieved with the help of many different ideas and processes such as improving toxins, improving injection techniques, but above all, new indications. When I look at my old lectures, I always find the distinction between indications already approved, indications currently being investigated in studies, and potential future indications.

Today, only the first topic remains: what has already been approved. That is why I am appealing to our young colleagues: Be courageous again! Show us where we are standing riveted to the spot, and what we have overseen. Pick up the leitmotiv of this unparalleled success story and continue its development. I am certain that my generation has either overlooked or insufficiently examined some indications, and that the success story of botulinum toxin is far from over yet. I invite you to join me on the final leg of my marathon and urge you to take up the baton from my generation and carry it forward, just as we took up the baton from the previous generation and carried it on.
